# Supplementary material for: Structure Elucidation of Triterpenoid Saponins Found in an Immunoadjuvant Preparation of Quillaja brasiliensis Using Mass Spectrometry and 1H and 13C NMR Spectroscopy
Source: Molecules. 2022 Apr 8;27(8):2402. doi: 10.3390/molecules27082402 (PMC9024837; doi:10.3390/molecules27082402)
Supplement: Supplementary file 1 [file molecules-27-02402-s001.zip › molecules-1668704-supplementary.pdf]

molecules

MDPI

Supplementary Materials

Structure elucidation of triterpenoid saponins found in immunoadjuvant preparation of *Quillaja brasiliensis* using mass spectrometry and ^{1}H and ^{13}C NMR spectroscopy.

Federico Wallace [1], Carolina Fontana [2,*], Fernando Ferreira [1,3] and Cristina Olivaro [1,*]

[1] Espacio de Ciencia y Tecnología Química, CENUR Noreste, Universidad de la República, Tacuarembó, Uruguay; federico.wallace@cut.edu.uy (F.W.), cristina.olivaro@cut.edu.uy (C.O.)
[2] Laboratorio de Espectroscopía y Fisicoquímica Orgánica, Departamento de Química del Litoral, CENUR Litoral Norte, Universidad de la República, Paysandú, Uruguay; cfontan@fq.edu.uy
[3] Laboratorio de Carbohidratos y Glicoconjugados, Departamento de Química Orgánica, Facultad de Química, Universidad de la República, Montevideo, Uruguay; ff@fq.edu.uy (F.F.)
* Correspondence: cfontan@fq.edu.uy (C.F.), cristina.olivaro@cut.edu.uy (C.O.)

Table S1. The ^{1}H and ^{13}C NMR chemical shifts (ppm) of the triterpene and fatty acyl chain moieties of saponin **Qb1**. 2

Table S2. The ^{1}H and ^{13}C NMR chemical shifts (ppm) for the triterpene and acyl chain moieties of saponin **S13**. 3

Table S3. ^{1}H and ^{13}C NMR chemical shifts (ppm) of the two oligosaccharide chains of the **S13** saponin, and inter-residue correlations from ^{1}H,^{1}H-NOESY and ^{1}H,^{13}C-HMBC spectra. The spectra were recorded in CD_3OD at 25°C on a Burker Avance 500 MHz spectrometer. 4

Figure S1. Selected regions of the ^{1}H,^{1}H-TOCSY spectrum of **Qb1** showing correlations from anomeric protons. 5

Figure S2. Selected region of the ^{1}H,^{13}C-HMBC spectrum of **Qb1** showing key correlations from anomeric protons. Only the inter-residue correlations are annotated. 6

Table S1. The ^{1}H and ^{13}C NMR chemical shifts (ppm) of the triterpene and fatty acyl chain moieties of saponin **Qb1.**

Residue	**Qb1**	
	δ_C	δ_H
Qa1	39.3	1.10, 1.70
Qa2	25.7	1.78, 1.97
Qa3	86.4	3.86
Qa4	56.3	-
Qa5	49.2	1.31
Qa6	21.5	0.93, 1.45
Qa7	33.6	1.31, 1.51
Qa8	41.2	-
Qa9	48.0	1.73
Qa10	37.1	-
Qa11	24.6	1.92
Qa12	123.2	5.33
Qa13	144.8	-
Qa14	42.8	-
Qa15	36.5	1.40, 1.66
Qa16	74.7	4.45
Qa17	50.3	-
Qa18	42.3	2.92
Qa19	48.2	1.05, 2.29
Qa20	31.3	-
Qa21	36.6	1.16, 1.93
Qa22	31.9	1.79, 1.90
Qa23	212.1	9.46
Qa24	11.0	1.16
Qa25	16.6	1.01
Qa26	18.0	0.77
Qa27	27.1	1.39
Qa28	177.3	-
Qa29	33.4	0.87
Qa30	25.0	0.95
Fa I-1	172.9	-
Fa I-2	44.0	2.57, 2.57
Fa I-3	66.1	4.02
Fa I-4	39.9	1.79
Fa I-5	75.3	5.19
Fa I-6	39.9	1.61
Fa I-7	26.6	1.16, 1.48
Fa I-8	12.3	0.91

Fa I-9	15.2	0.91
Fa II-1	173.7	-
Fa II-2	44.0	2.47, 2.47
Fa II-3	66.5	4.31
Fa II-4	39.4	1.49, 1.61
Fa II-5	80.2	3.81
Fa II-6	40.3	1.6
Fa II-7	25.1	1.08, 1.69
Fa II-8	12.5	0.91
Fa II-9	14.8	0.92

Table S2. The ^{1}H and ^{13}C NMR chemical shifts (ppm) for the triterpene and acyl chain moieties of saponin **S13**.

N°	**S13**	
	δ_C	δ_H
Pa1	39.4	0.98, 1.63
Pa2	26.4	1.75, 2.1
Pa3	84.0	3.59
Pa4	43.3	
Pa5	48.9	1.08
Pa6	19.2	1.31, 1.35
Pa7	33.3	1.29, 1.39
Pa8	40.9	
Pa9	49.2	1.59
Pa10	37.8	
Pa11	24.4	1.92, 1.92
Pa12	124.0	5.32
Pa13	144.5	
Pa14	42.8	
Pa15	29.4	1.25, 1.54
Pa16	23.9	1.68, 2.06
Pa17	47.8	
Pa18	44.1	2.69
Pa19	43.2	1.70, 1.96
Pa20	45.0	
Pa21	31.2	1.40, 2.04
Pa22	34.3	1.59, 1.69
Pa23	66.7	4.00, 4.25
Pa24	13.2	0.82
Pa25	16.6	0.99
Pa26	17.7	0.81
Pa27	25.8	1.14

Pa28	178.0	
Pa29	28.4	1.14
Pa30	178.8	
OMe	52.2	3.71
Fa I-1	178.6	
Fa I-2	42.6	2.52
Fa I-3	27.9	1.54, 1.74
Fa I-4	12.1	0.99
Fa I-5	17.3	1.19
Fa II-1	177.7	
Fa II-2	42.2	2.32
Fa II-3	27.6	1.47, 1.68
Fa II-4	12.0	0.93
Fa II-5	16.6	1.11
Ac I-1	172.2	
Ac I-2	20.9	2.08
Ac II-1	173.0	
Ac II-2	20.8	2.05

Table S3. ^{1}H and ^{13}C NMR chemical shifts (ppm) of the two oligosaccharide chains of the **S13** saponin, and inter-residue correlations from $^{1}H,^{1}H$-NOESY and $^{1}H,^{13}C$-HMBC spectra. The spectra were recorded in CD_3OD at 25 °C on a Burker Avance 500 MHz spectrometer.

Residue	^{1}H / ^{13}C						Correlation to atom (from anomeric atom)	
	1	2	3	4	5	6	HMBC	NOESY
Pa C3-O-glycan								
→2)-β-D-Glc*p*A-(1→	4.37 [7.6]	3.50	3.57	3.53	3.65		C3, **Pa**[(a)]	H3, **Pa**[(a)]
	104.9	82.8	78.0	76.4	76.8	173.8		
β-D-Gal*p*-(1→	4.55 [7.7]	3.55	3.48	3.83	3.46	3.7, 3.7	C2, **GlcA**	H2, **GlcA**
	106.0	73.7	74.7	69.9	76.6	61.7		
Pa C28-O-glycan								
→2,3,4)-β-D-Fuc*p*-(1→	5.47 [8.2]	3.97	4.12	5.38	3.90	1.05	C28, **Pa**[(a)]	
	94.8	73.2	82.6	74.7	70.9	16.4		
→2,3)-α-L-Rha*p*-(1→	5.57 [1.5]	5.34	4.98	3.51	3.94	1.29	C2, **Fuc**	H2, **Fuc**
	98.6	70.9	72.9	70.7	70.1	17.8		
β-D-Glc*p*-(1→	4.48 [7.6]	3.04	3.31	3.17	3.29	3.62, 3.86	C3, **Fuc**	H3, **Fuc**
	105.2	75.1	77.6	71.2	77.5	62.8		

$^{3}J_{H1,H2}$ values are given in hertz in square brakets. [(a)] Chemical shifts of these atoms are shown in Table S2.

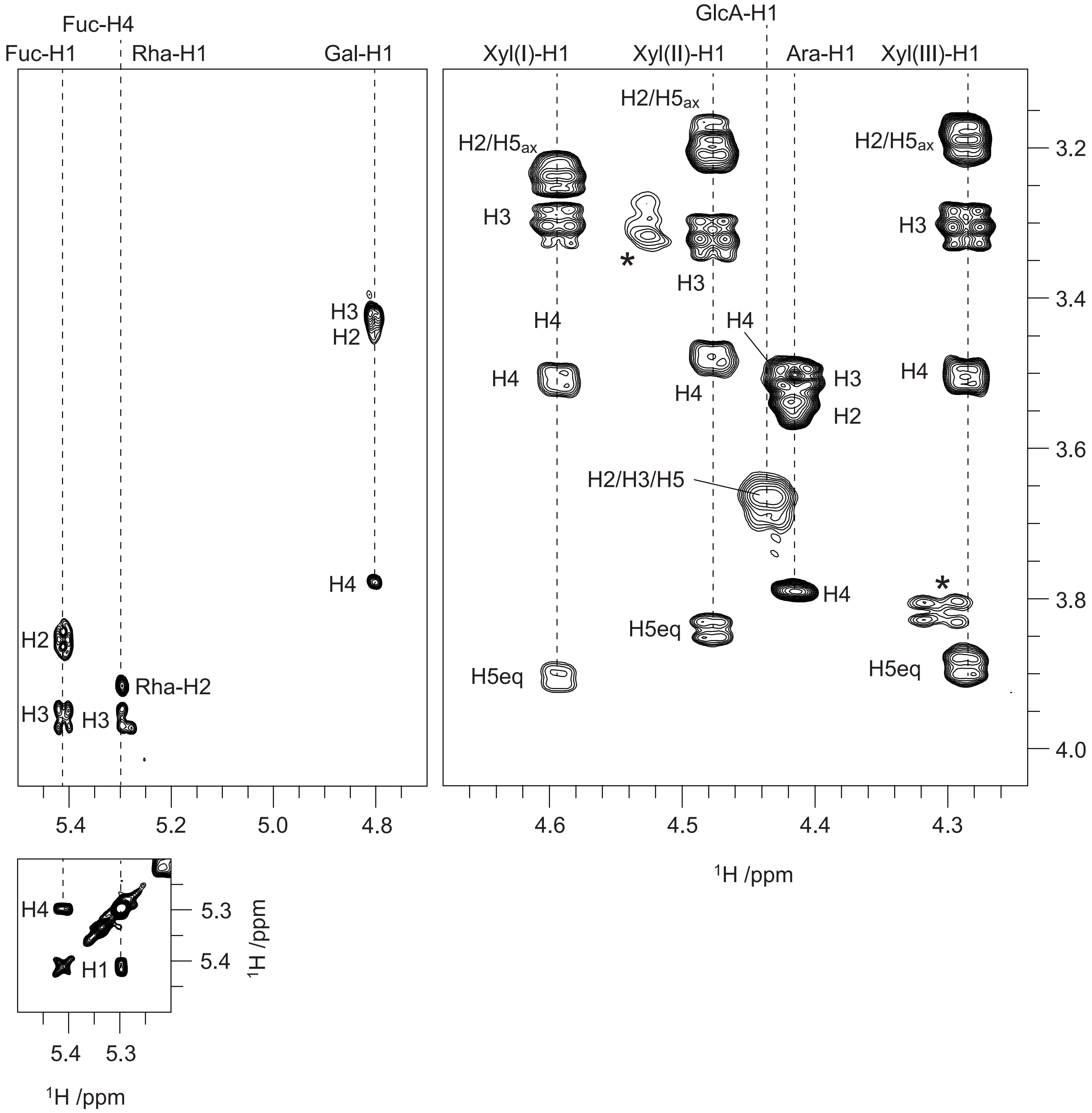

Figure S1. Selected regions of the $^1H,^1H$-TOCSY spectrum of **Qb1** showing correlations from anomeric protons.

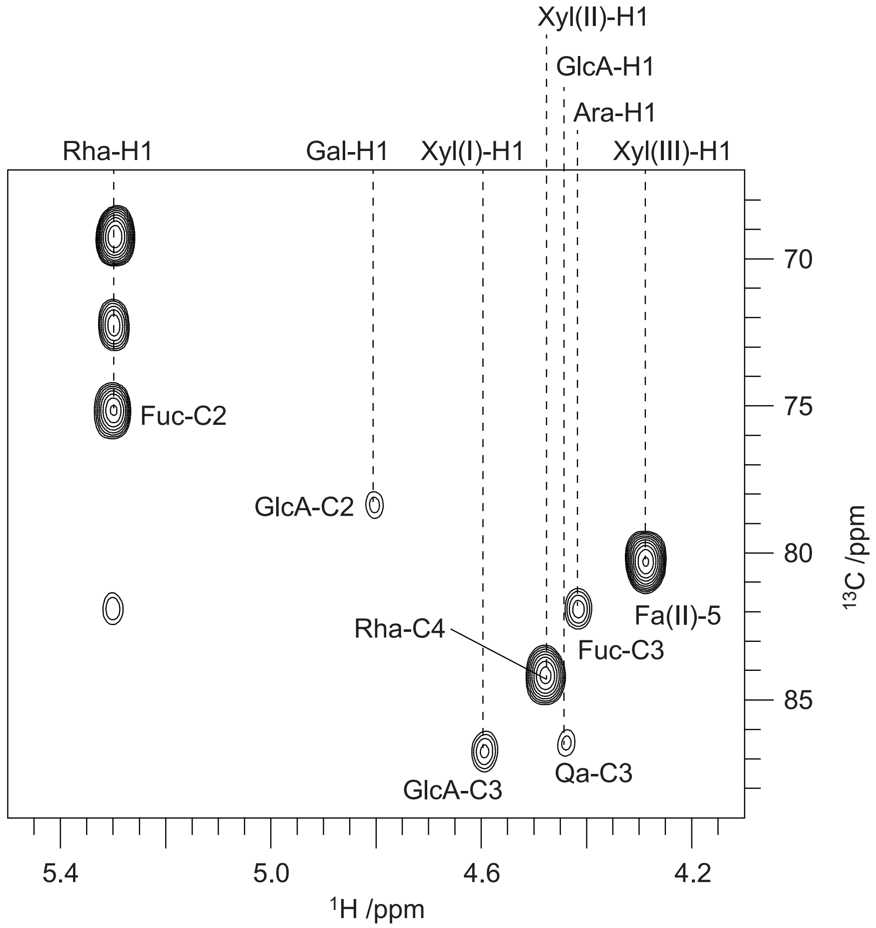

Figure S2. Selected region of the ^1H,^{13}C-HMBC spectrum of **Qb1** showing key correlations from anomeric protons. Only the inter-residue correlations are annotated.
